# Supplementary material for: Structure of the endocytic adaptor complex reveals the basis for efficient membrane anchoring during clathrin-mediated endocytosis
Source: Nat Commun. 2021 May 17;12:2889. doi: 10.1038/s41467-021-23151-7 (PMC8129110; doi:10.1038/s41467-021-23151-7)
Supplement: Supplementary file 1 — Supplementary Information [file 41467_2021_23151_MOESM1_ESM.pdf]

Supplementary Information

Structure of the endocytic adaptor complex reveals the basis for efficient membrane anchoring during clathrin-mediated endocytosis

Javier Lizarrondo[1], David P. Klebl[2], Stephan Niebling[1], Marc Abella[3], Martin A. Schroer[1], Haydyn D.T. Mertens[1], Katharina Veith[1], Roland Thuenauer[4], Dmitri I. Svergun[1], Michal Skruzny[3], Frank Sobott[5,6], Stephen P. Muench[2] and Maria M. Garcia-Alai[1,7]

1 European Molecular Biology Laboratory, Hamburg Outstation, Hamburg, Germany.

2 School of Biomedical Sciences, Faculty of Biological Sciences & Astbury Centre for Structural and Molecular Biology, University of Leeds, Leeds, UK. School of Biomedical Sciences, Faculty of Biological Sciences, University of Leeds, Leeds, UK.

3 Department of Systems and Synthetic Microbiology, Max Planck Institute for Terrestrial Microbiology and LOEWE Center for Synthetic Microbiology (SYNMIKRO), Marburg, Germany

4 Technology Platform Microscopy and Image Analysis, Heinrich Pette Institute, Leibniz Institute for Experimental Virology, Hamburg, Germany

5 School of Molecular and Cellular Biology, Faculty of Biological Sciences & Astbury Centre for Structural and Molecular Biology, University of Leeds, Leeds, UK.

6 Department of Chemistry, Biomolecular & Analytical Mass Spectrometry group, University of Antwerp, Antwerp, Belgium.

7 Centre for Structural Systems Biology, Notkestrasse 85, D-22607 Hamburg, Germany.

Lizarrondo et. al., 2021

Javier Lizarrondo and David P. Klebl contributed equally to this work. Correspondence and requests for materials should be addressed to M.G.A. (email: garcia@embl-hamburg.de).

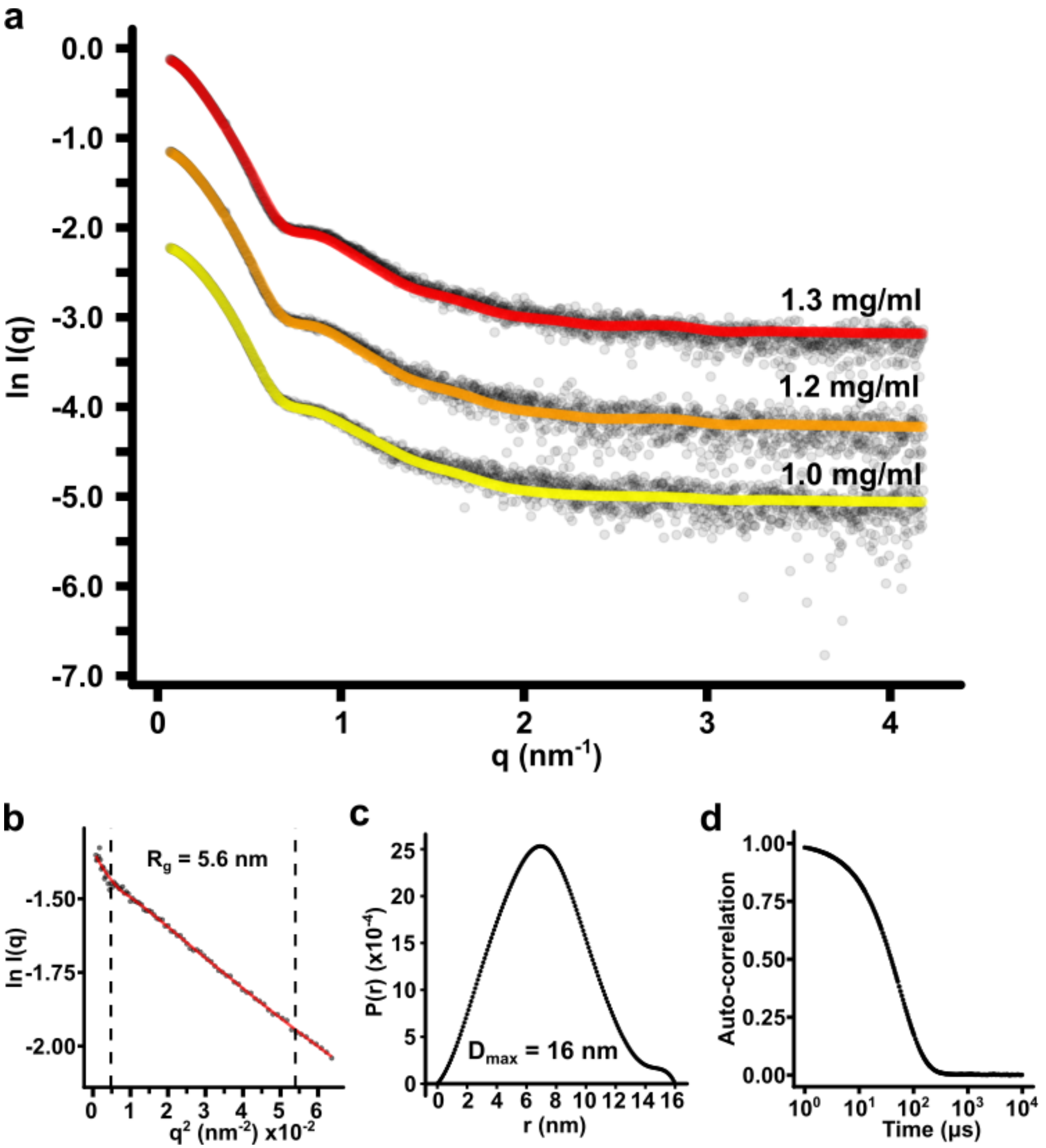

Supplementary Fig. 1. **Biophysical characterization of the AENTH assembly in solution**. **a.** Experimental SAXS data of the 16-mer assembly (A_8E_8) in presence of 200 μM PIP_2. The red, orange and yellow lines represent the fittings from OLIGOMER using a mixture population of monomers (ANTH & ENTH) and oligomers (16-mer and 32-mer). The contributions of monomer, 16-mer and 32-mer are: 0.09, 0.39, 0.52 for the curve at 1.3 mg/ml (χ^2 =3.81); 0.08, 0.47, 0.42 for the curve at 1.2 mg/ml (χ^2 =1.81); and

0.15, 0.58, 0.27 for the curve at 1 mg/ml (χ^2 =1.44). **b.** Guinier plot for calculation of the R_g of the SAXS curves. **c.** Distance-distribution function obtained from the SAXS curves. **d.** DLS auto-correlation curve for the sample used for cryo-EM. The approximate hydrodynamic radius of the sample was 9 nm.

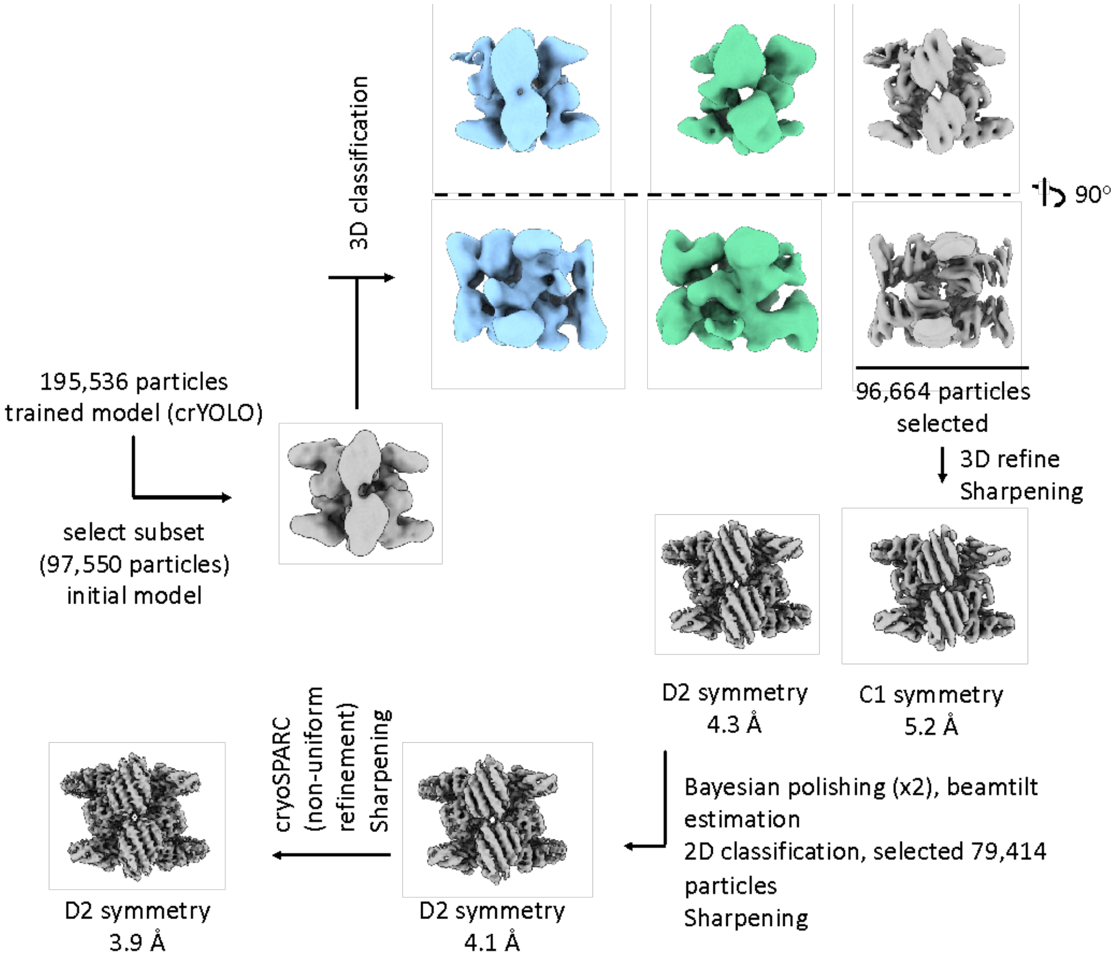

Supplementary Fig. 2. Processing flowchart for the 16-mer AENTH complex.

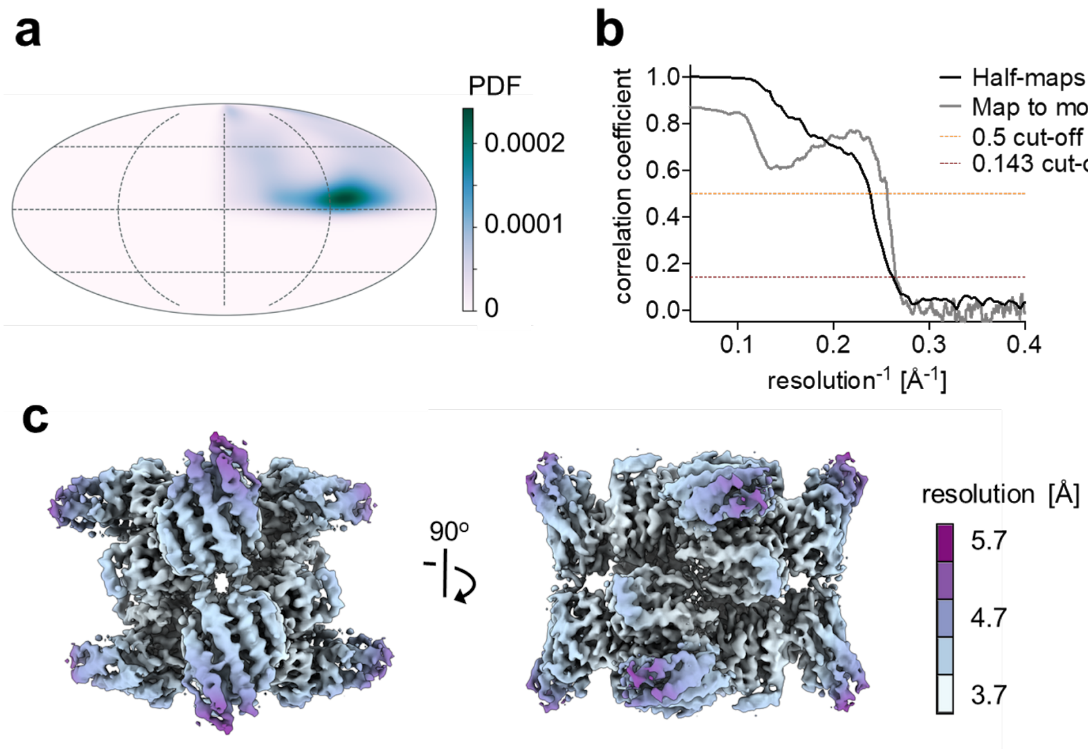

Supplementary Fig. 3. Cryo-EM reconstruction. a. Angular distribution (PDF = Probability density function). **b.** Fourier Shell Correlation (FSC) curves and **c.** local resolution for the AENTH 16-mer complex.

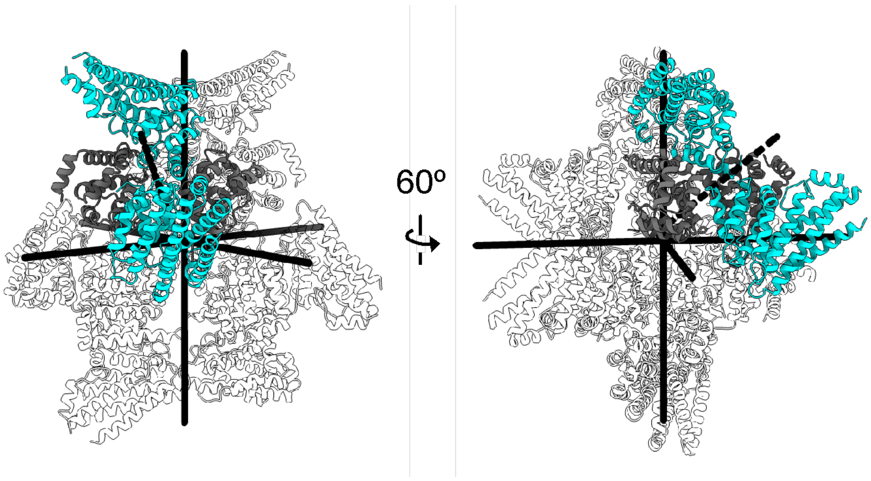

Supplementary Fig. 4. Symmetry of the 16-mer complex. Structure of 16-mer AENTH (A_8E_8) complex with symmetry axes indicated by black solid lines. ANTH and ENTH subunits of one asymmetric unit (tetramer) are shown in cyan and grey, respectively. The pseudo-2fold axis within the tetramer is shown as dashed black line.

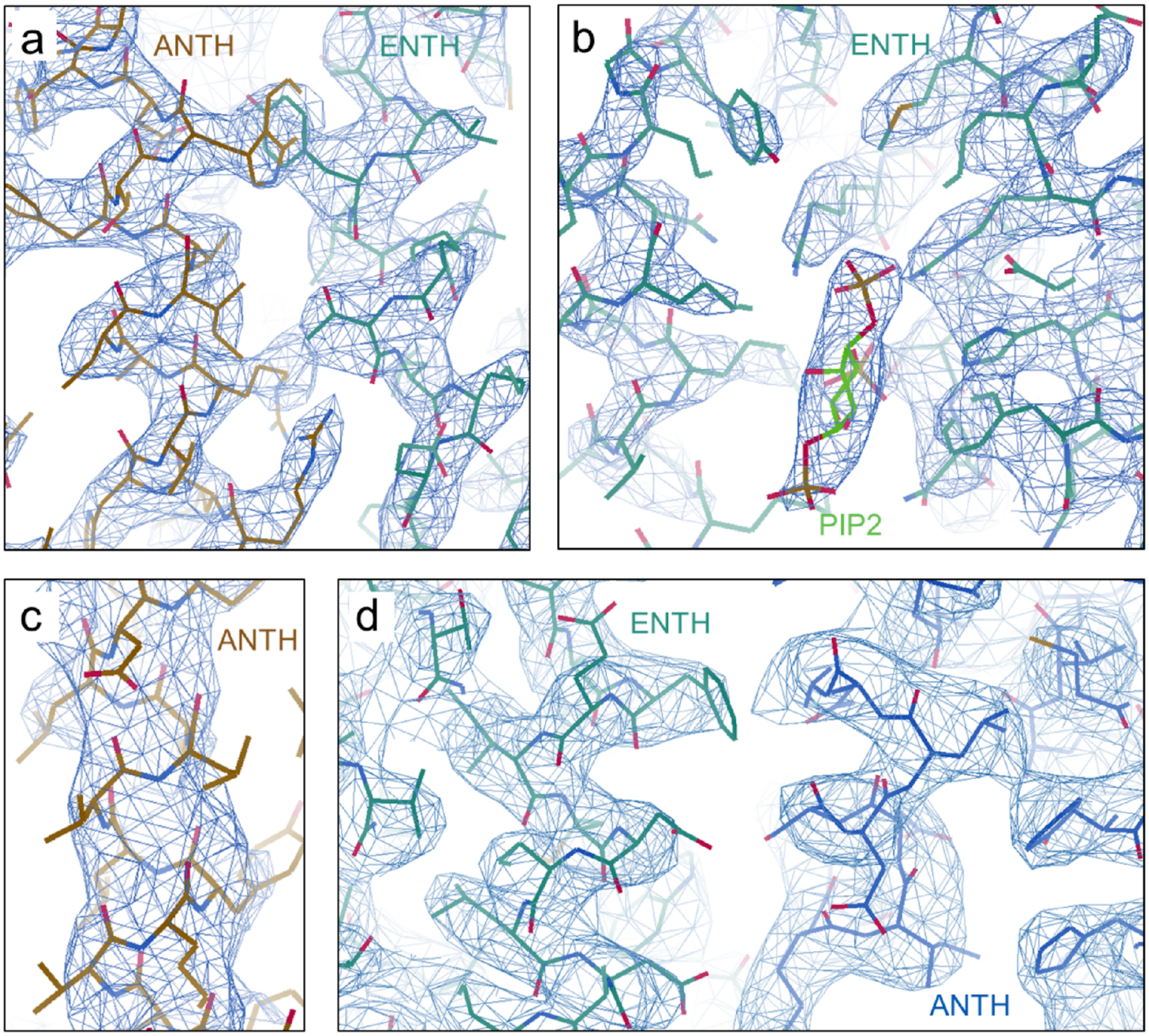

Supplementary Fig. 5. Representative EM densities. a. Well-resolved central alpha-helical parts of the ANTH-ENTH complex. **b**. One of the PIP_2-binding sites between two ENTH domains **c**. Lower resolution at the periphery of the complex in the last alpha helix of the ANTH domain and (**d**) the ANTH-ENTH interface 2.

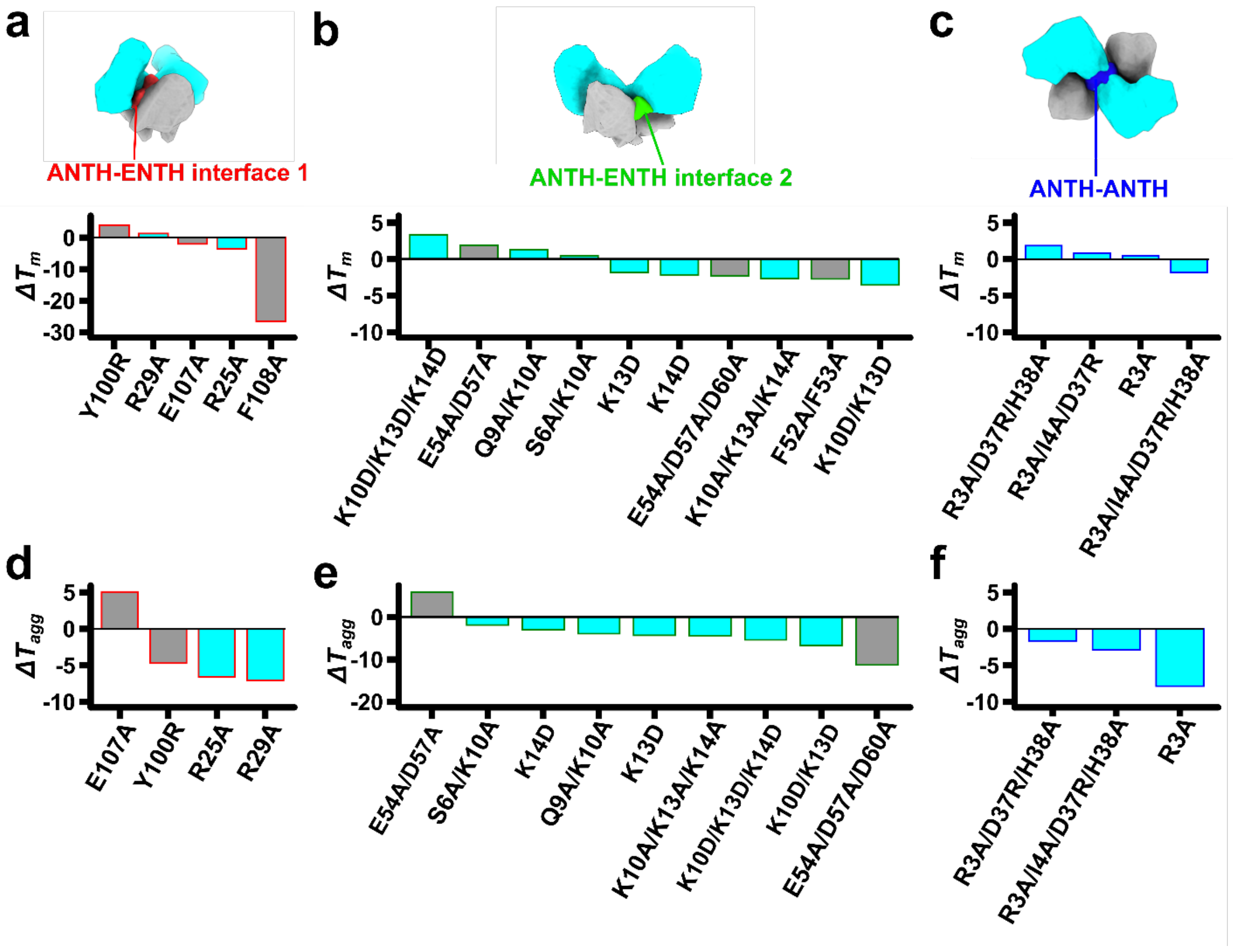

Supplementary Fig. 6. nanoDSF data for the ANTH and ENTH mutants affecting ANTH-ENTH interface 1 (red), ANTH-ENTH interface 2 (green) and ANTH-ANTH interface (blue) (**a, b and c**): Difference in melting temperature (T_m) between mutant domains compared to ANTH wt (cyan) or ENTH wt (grey). The T_m was obtained from the fluorescence signal ratio 350/330 nm. All domains with a ΔT_m higher than 2 °C were not further considered for complex assembly experiments. (**d, e and f**): Difference in the mid-aggregation temperature (T_{agg}) from AENTH complexes assembled with different mutant domains (ANTH mutant + ENTH wt, cyan and ENTH mutant + ANTH wt, grey) compared to AENTH wt. T_{agg} was obtained from the scattering signal of the nanoDSF melting experiments. Most mutants showed a lower aggregation temperature when compared with wild-type complex.

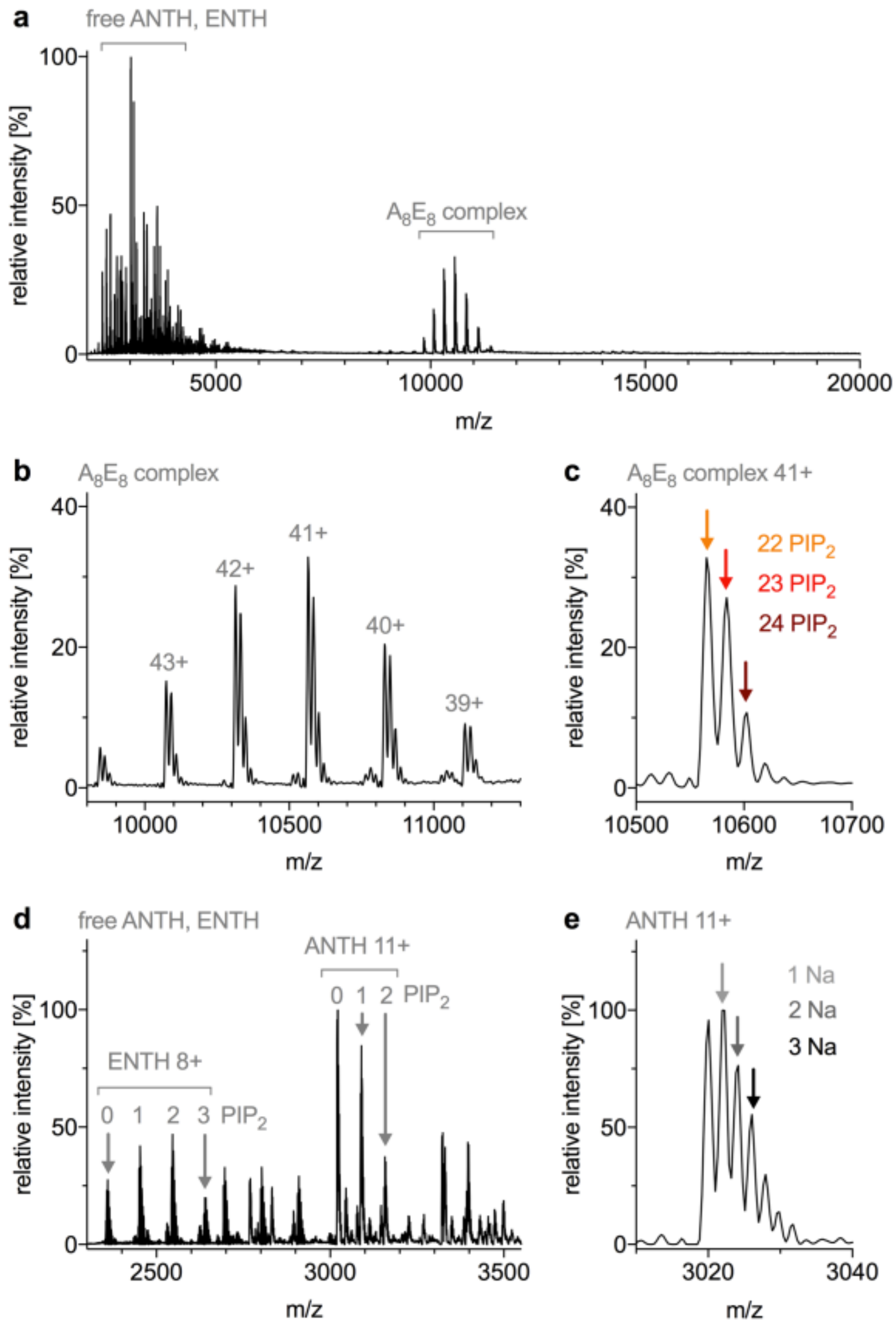

Lizarrondo et. al., 2021

Supplementary Fig 7. Native MS of the AENTH WT complex in the presence of 60 µM PIP_2. a. Full m/z range of the spectrum. The presence of free ANTH and ENTH is observed at low m/z while the complex appears around 10500 m/z. **b.** Charge state distribution of the A_8E_8 8:8 ANTH/ENTH complex with individual charge states labelled **c.** Close up of the 41+ charge state of the A_8E_8 8:8 ANTH/ENTH complex showing different numbers of PIP_2 molecules bound to the complex (22, 23 and 24 are clearly distinguishable). **d.** Close up of the spectrum at lower m/z where ENTH bound to 0-3 PIP_2 molecules and ANTH bound to 0-2 PIP_2 molecules can be observed. **e.** Peak fine structure of the ANTH (no PIP2) 11+ charge state shows adducts with 1, 2 and 3 Na^+ molecules.

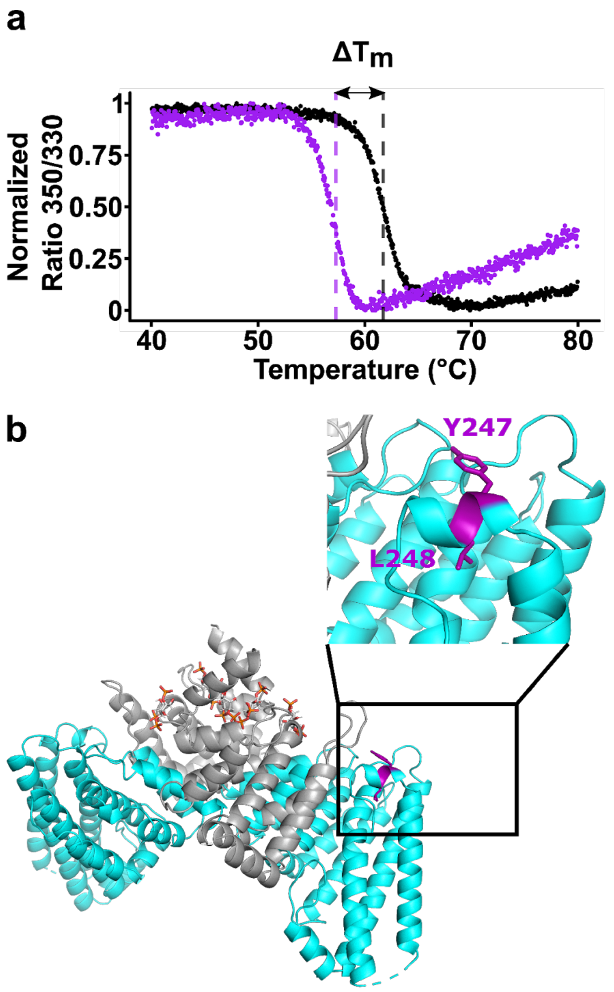

Supplementary Fig. 8. Sla2 Y247-L248 insertion is not part of the AENTH interface. a. nanoDSF data for ANTH wt (black, T_m= 61.7 °C) and ANTH ΔY247/L248 (violet, T_m = 57.7 °C). The shift in the transition of the nanoDSF signal can be clearly observed and is indicated as ΔT_m, indicating that this mutation destabilizes the protein. The T_m was obtained from the fluorescence signal ratio 350/330 nm. **b.** Residues Y247 and L248 of ANTH domain did not establish any contacts in the AENTH complex. Cartoon representation of the AENTH tetramer and the PIP_2 molecules bound to it, with the YL residues colored in violet. The insert shows the detailed orientation of these residues, which does not establish any protein-protein contacts. Deletion of these residues disrupts helix α12.

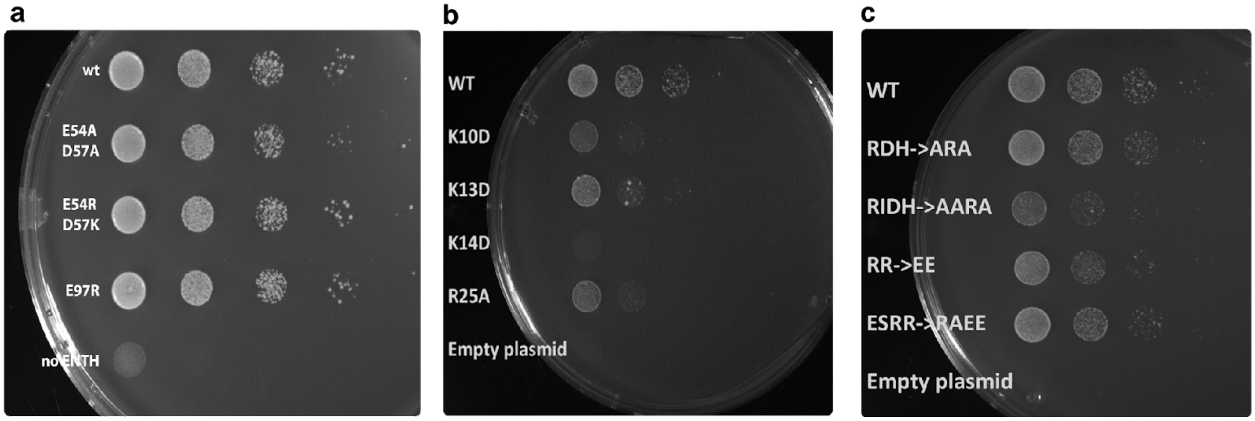

Supplementary Fig. 9. Growth defects of selected mutants affecting the ANTH-ENTH interface 2 (**a** and **b**) and the ANTH-ANTH interface (**c**). ANTH wt and ENTH wt domains or indicated interface mutants were expressed after depletion or deletion of endogenous Ent1 and Sla2 proteins, respectively. Cell growth was analysed after plating 10-fold serial dilution of cells on SD-Ura plates and incubation for 3 days at 37 °C. **a.** Mutation of negatively charged residues ENTH E54 and D57 does not introduce a growth defect. **b.** Mutation of single ANTH positively charged residues K10, K13, K14 and R25 impair cell growth. **c.** Growth of mutants of the ANTH-ANTH interface (RDH->ARA, R3A/D37R/H38A, RIDH-> AARA, R3A/I4A/D37R/H38A) and the ANTH loop 175-183 (RR->EE, R178E/E178E, ESRR-> RAEE, E57R/S100A/R178E/E178E).

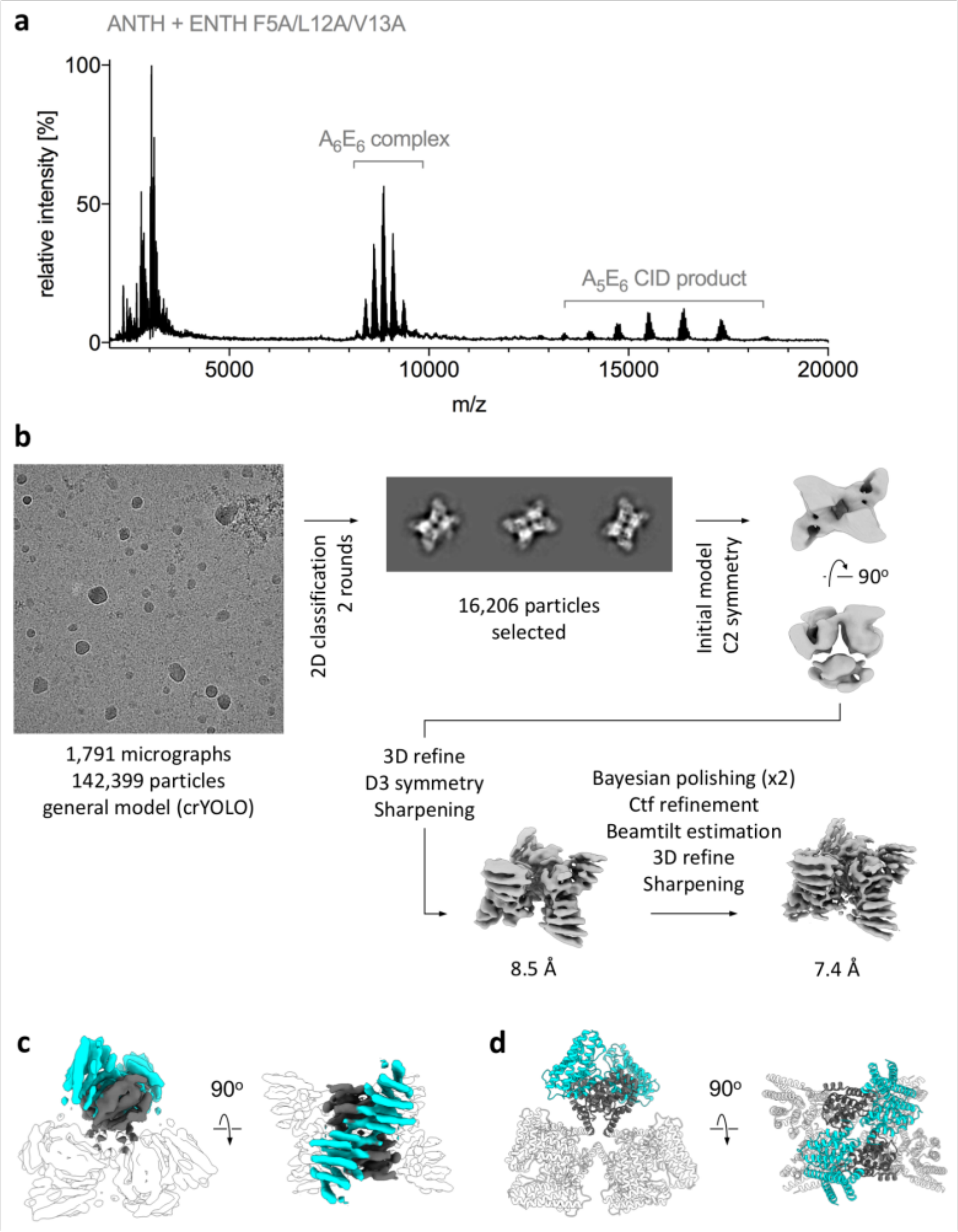

Supplementary Fig. 10. **AENTH 12-mer assembly (A_6E_6) obtained by mutation of residues F5A/L12A/V13A of the amphipatic α0 helix of the ENTH domain**. **a.** Native MS of ANTH in complex

with ENTH F5A/L12A/V13A at 200 µM PIP_2. The main oligomeric species is the 6:6 AENTH complex. At high m/z, a 5:6 ANTH/ENTH complex is present, resulting from collision induced dissociation (CID) of the 6:6 complex. **b.** Processing flowchart for the 12-mer AENTH complex. **c**. Final density map obtained for the 12-mer assembly with one tetramer coloured in cyan for the ANTH domain and in grey for the ENTH domain. **d**. Structural model for the 12-mer assembly. The tetramer structure (A_2E_2) was fitted into the EM density map for each of the three tetramers. The ANTH and ENTH domains are coloured for one of the three tetramers in the same colour code as in **c**.

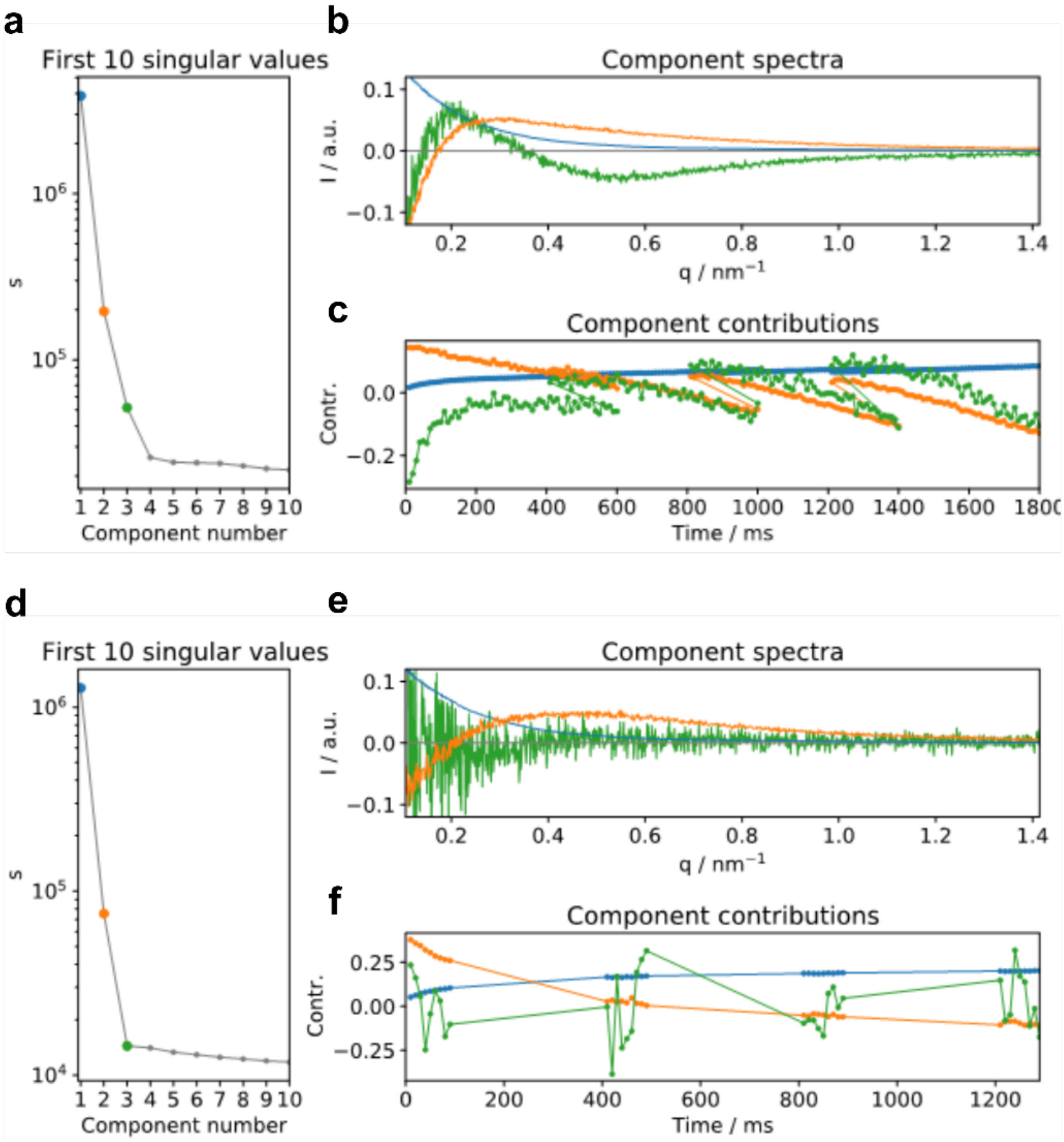

Supplementary Fig 11. SVD analysis of SF-TR-SAXS. a. SVD analysis of the full dataset revealed 3 components present. **b.** SAXS spectra of the three components. **c.** Component contribution over time of the SVD analysis using the full dataset. **d.** SVD analysis of the dataset using the first 10 points after beam exposure reveals two major components. **e.** SAXS spectra of the three first components. **f.** Component contributions over time of the SVD analysis using the first 10 points after beam exposure.

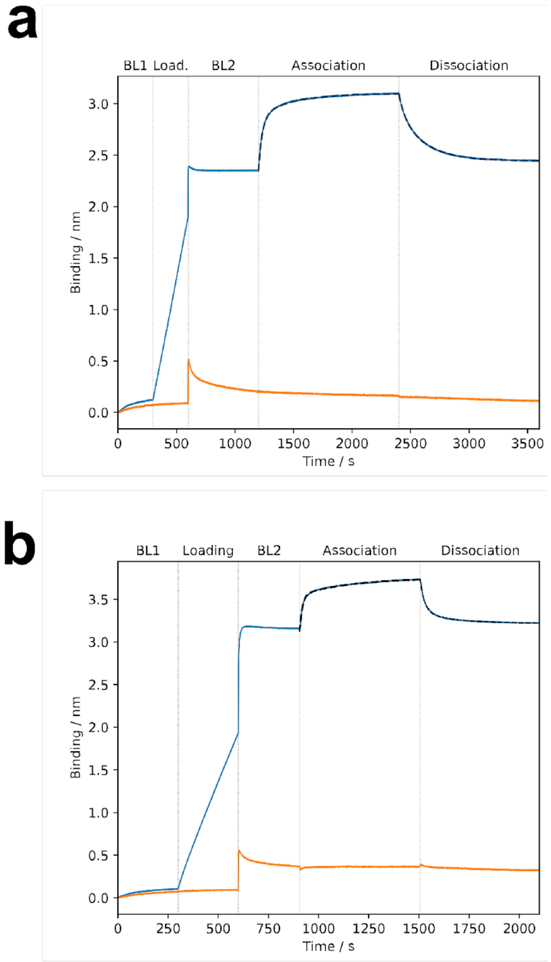

Supplementary Fig. 12. AENTH complex formation is a reversible process. Binding kinetics measured by biolayer interferometry (BLI) between His-tagged ANTH (**a**) or ENTH (**b**) immobilized on a Ni-NTA Octet sensor (load stage performed at 3.7 µg/ml monomeric protein) in the presence of 0.25 µM free ENTH (**a**) or ANTH (**b**) (blue curves) and without ligand (orange curves). All steps were done in 50 mM Tris HCl pH 8.0, 125 mM NaCl and 0.05% BSA. For baseline 2 (BL2), association and dissociation stages the buffer additionally contained 170 µM DDM and 50 µM PIP_2. See Table 3 for the kinetic constants.

Supplementary Table 1. ANTH-ENTH complexes measured by DLS

ANTH	ENTH	Radius (nm)	%Pd	Mw-R (kDa)	%Intensity	%Mass	%Number
WT	WT	9.6	11.85	664.6	100	100	0
R25A	WT	2.9	8.85	41.9	1.8	64.0	99.0
		8.2	8.56	468.1	9.7	15.2	1.0
R29A	WT	2.5	15.06	29.3	11.1	90.6	99.6
		7.2	14.29	343.9	22.2	7.7	0.4
WT	Y100R	2.7	0	33.3	15.2	78.5	98.1
		7.2	14.81	344.4	71.3	21.3	1.9
WT	E107A	3.1	4.61	47.2	3.2	29.8	78.0
		7.1	16.06	331.7	76.6	68.2	22.0
WT	E54A/D57A/D60A	7.5	26.06	374	100	100	100
S6A/K10A	WT	6.7	6.86	287.7	93.8	99.4	100
K13D	WT	7.2	10.19	344.6	100	100	100
Q9A/K10A	WT	8	11.18	433.9	96.8	99.2	100
K10A/K13A/K14A	WT	8.5	16.31	508	90.7	28.4	1.6
WT	E54A/D57A	3.3	11.49	55.8	3.5	73.1	99.5
		9.7	8.91	684.9	9.5	8.2	0.5
K10D/K13D/K14D	WT	3.2	12.67	51.4	39.8	99.9	100
R3A	WT	2.5	9.08	28.9	9.9	79.1	99.3
		8.1	6.85	454.0	89.0	20.8	0.7
R3A/I4A/D37A	WT	8.3	9.15	479.8	100	100	100
R3A/D37R/H38A	WT	7.5	9.83	380.4	94.8	99.2	100.0
K10D/K13D	WT	7.5	40.8	381.3	100	100	100
K14D	WT	7.4	24.5	358.9	89	99	100
WT	F5A/I12A/V13A	8	11.62	436	100	100	100
ΔY247/L248	WT	9	36.27	571.8	100	100	100

Supplementary Table 2: Data collection and processing parameters for the AENTH 12-mer assembly (ANTH WT, ENTH F5A/L12A/V13A).

**Data collection and processing**	
Magnification	×75,000
Voltage (kV)	300
Electron exposure ($e^{-}/Å^2$)	72.6
Defocus range (µm)	-1.5 to -4.2
Pixel size (Å)	1.065
Symmetry imposed	D_3
Initial particle images (no.)	142,399
Final particle images (no.)	16,206
Map resolution (Å)	7.4
FSC threshold	0.143
Map resolution range (Å)	6.8 to 9.9

Supplementary Table 3. Fitting for the binding kinetics determined by BLI

Figure S11 A		
**Fit rise**	**Fit decay**	
k_{obs1} : 3.06E^{-02} 1/s	k_{diss1} : 4.40E^{-03} 1/s	K_{D1} : 3.14E-08 M
k_{obs2} : 3.45E^{-03} 1/s	k_{diss2} : 1.91E^{-02} 1/s	K_{D2} : 2.12E^{-07} M
R^2 : 0.9989	R^2 : 0.9995	
**Figure S11 B**		
**Fit rise**	**Fit decay**	
k_{obs1} : 7.45E^{-02} 1/s	k_{diss1} : 6.23E^{-03} 1/s	K_{D1} : 1.93E^{-08}M
k_{obs2} : 3.87E^{-03} 1/s	k_{diss2} : 4.56E^{-02} 1/s	K_{D2} : 2.30E^{-07}M
R^2 : 0.9980	R^2 : 0.9981	

Supplementary Table 4. Primers used to produce ANTH and ENTH mutants.

PCR primers			
	Mutant	Forward	Reverse
ANTH	M1A	cagggcgccatggGATCCgcgAGCCGTATCGATTCTGACCTGCAG	CTGCAGGTCAGAATCGATACGGCTcgcGGATCccatggcgccctg
ANTH	S6A/K10A	catggGATCCATGAGCCGTATCGATgCgGACCTGCAGgcgGCGCTCAAAAAAGCCTGTTCCGTTG	CAACGGAACAGGCTTTTTTGAGCGCcgcCTGCAGGTCcGcATCGATACGGCTCATGGATCccatg
ANTH	Q9A/K10A	CCATGAGCCGTATCGATTCTGACCTGgcggccGCGCTCAAAAAAGCCTGTTCCGTTGAGG	CCTCAACGGAACAGGCTTTTTTGAGCGCggccgcCAGGTCAGAATCGATACGGCTCATGG
ANTH	K13D	GATTCTGACCTGCAGAAAGCGCTCgatAAAGCCTGTTCCGTTGAGGAAACCG	CGGTTTCCTCAACGGAACAGGCTTTatcGAGCGCTTTCTGCAGGTCAGAATC
ANTH	R3A/I4A	ctttattttcagggcgccatggGATCCATGAGCgcggccGATTCTGACCTGCAGAAAGCGCTC	CGGTGCGGTTTCCTCAACGGAACAGGCcgcggcGAGCGCggcCTGCAGGTCAGAATCGATACGGC
ANTH	ΔY247/L248	GTTTTACGCGGATTGCTCCTCTGTGAAAACCACGCTGGTTACCATTCCAAAACTGC	GCAGTTTTGGAATGGTAACCAGCGTGGTTTTCACAGAGGAGCAATCCGCGTAAAAC
ANTH	K10A/K13A/K14A	GCCGTATCGATTCTGACCTGCAGgccGCGCTCgccgcgGCCTGTTCCGTTGAGGAAACCGCACCG	CGGTGCGGTTTCCTCAACGGAACAGGCcgcggcGAGCGCggcCTGCAGGTCAGAATCGATACGGC
ANTH	K10D/K13D/K14D	GCCGTATCGATTCTGACCTGCAGgacGCGCTCgatgacGCCTGTTCCGTTGAGGAAACCGCACCG	CGGTGCGGTTTCCTCAACGGAACAGGCgtcatcGAGCGCgtcCTGCAGGTCAGAATCGATACGGC
ANTH	K10D/K13D	CTGACCTGCAGgacGCGCTCgatAAAGCCTGTTCCGTTGAGGAAACCG	CGGTTTCCTCAACGGAACAGGCTTTatcGAGCGCgtcCTGCAGGTCAG
ANTH	K14D	CTGACCTGCAGAAAGCGCTCAAAgacGCCTGTTCCGTTGAGGAAACCGC	GCGGTTTCCTCAACGGAACAGGCgtcTTTGAGCGCTTTCTGCAGGTCAG
ANTH	R3A	cagggcgccatggGATCCATGAGCgcgATCGATTCTGACCTGCAGAAAGC	GCTTTCTGCAGGTCAGAATCGATcgcGCTCATGGATCccatggcgccctg
ANTH	R25A	GTTGAGGAAACCGCACCGAAAgcgAAACACGTACGTGCATGCATTGTGTAC	GTACACAATGCATGCACGTACGTGTTTcgcTTTCGGTGCGGTTTCCTCAAC
ANTH	R29A	CCGCACCGAAACGCAAACACGTAgcgGCATGCATTGTGTACACCTGGG	CCCAGGTGTACACAATGCATGCcgcTACGTGTTTGCGTTTCGGTGCGG
ENTH	E54A/D57A/D60A	CTACGACAGCGCAGACTTCTTTGcGATCATGGcgATGCTGGcgAAACGCCTGAACGACAAAGGC	GCCTTTGTCGTTCAGGCGTTTcgCCAGCATcgCCATGATCgCAAAGAAGTCTGCGCTGTCGTAG
ENTH	Y100R	GTACTGTGGTGCCGTGAGAACCTGcgtATCATCAAAACCCTGAAAGAG	CTCTTTCAGGGTTTTGATGATacgCAGGTTCTCACGGCACCACAGTAC
ENTH	F108A	CATCATCAAAACCCTGAAAGAGgcgCGTCACGAAGACGATGAGGGTATTG	CAATACCCTCATCGTCTTCGTGACGcgcCTCTTTCAGGGTTTTGATGATG
ENTH	E107A	GTACATCATCAAAACCCTGAAAGcGTTCCGTCACGAAGACGATGAGGG	CCCTCATCGTCTTCGTGACGGAACgCTTTCAGGGTTTTGATGATGTAC
ENTH	E54A/D57A	CGACAGCGCAGACTTCTTTGcGATCATGGcgATGCTGGACAAACGCCTGAAC	GTTCAGGCGTTTGTCCAGCATcgCCATGATCgCAAAGAAGTCTGCGCTGTCG
ENTH	F52A/F53A	GAAAAGCTACGACAGCGCAGACgcggcgGAGATCATGGACATGCTGGAC	GTCCAGCATGTCCATGATCTCcgccgcGTCTGCGCTGTCGTAGCTTTTC
ENTH	F5A/L12A/V13A	ccatggGATCCATGTCTAAACAGgcgGTGCGTTCCGCGAAAAACgcGGcGAAAGGTTATTCCTCTACCCAGGTAC	GTACCTGGGTAGAGGAATAACCTTTCgCCgcGTTTTTCGCGGAACGCACcgcCTGTTTAGACATGGATCccatgg
